# Utilizing ChatGPT-3.5 to Assist Ophthalmologists in Clinical Decision-making

**DOI:** 10.18502/jovr.v20.14692

**Published:** 2025-05-05

**Authors:** Samir Cayenne, Natalia Penaloza, Anne C. Chan, M.I. Tahashilder, Rodney C. Guiseppi, Touka Banaee

**Affiliations:** ^1^John Sealy School of Medicine, The University of Texas Medical Branch, Galveston, TX, USA; ^2^Department of Ophthalmology & Visual Sciences, The University of Texas Medical Branch, Galveston, TX, USA; ^3^Office of Biostatistics, The University of Texas Medical Branch, Galveston, TX, USA

**Keywords:** Artificial Intelligence, ChatGPT, Cornea, Neuro-ophthalmology, Retina

## Abstract

**Purpose:**

ChatGPT-3.5 has the potential to assist ophthalmologists by generating a differential diagnosis based on patient presentation.

**Methods:**

One hundred ocular pathologies were tested. Each pathology had two signs and two symptoms prompted into ChatGPT-3.5 through a clinical vignette template to generate a list of four preferentially ordered differential diagnoses, denoted as Method A. Thirty of the original 100 pathologies were further subcategorized into three groups of 10: cornea, retina, and neuro-ophthalmology. To assess whether additional clinical information affected the accuracy of results, these subcategories were again prompted into ChatGPT-3.5 with the same previous two signs and symptoms, along with additional risk factors of age, sex, and past medical history, denoted as Method B. A one-tailed Wilcoxon signed-rank test was performed to compare the accuracy between Methods A and B across each subcategory (significance indicated by *P* < 0.05).

**Results:**

ChatGPT-3.5 correctly diagnosed 51 out of 100 cases (51.00%) as its first differential diagnosis and 18 out of 100 cases (18.00%) as a differential other than its first diagnosis. However, 31 out of 100 cases (31.00%) were not included in the differential diagnosis list. Only the subcategory of neuro-ophthalmology showed a significant increase in accuracy (*P* = 0.01) when prompted with the additional risk factors (Method B) compared to only two signs and two symptoms (Method A).

**Conclusion:**

These results demonstrate that ChatGPT-3.5 may help assist clinicians in suggesting possible diagnoses based on varying complex clinical information. However, its accuracy is limited, and it cannot be utilized as a replacement for clinical decision-making.

##  INTRODUCTION

ChatGPT-3.5 is an artificial intelligence (AI) program designed to enact an instruction in a prompt and generate a detailed response. Given its novelty and adaptability to various contexts, including medicine, the reliability of ChatGPT in healthcare settings is largely unknown. A small but increasing number of studies have assessed its accuracy in clinical applications. Accordingly, ChatGPT may have the potential to assist physicians in clinical decision-making by analyzing large databases and, thus, proposing a clinical diagnosis.

Previous studies have shown that ChatGPT achieved an accuracy of 71.7% in diagnosing common chief complaints using ophthalmological and non-ophthalmological clinical vignettes from the Merck Sharp & Dohme (MSD) Clinical Manual. This finding reflects its acceptable accuracy (95% CI: 69.3–74.1%) when prompted with a vignette of complex cases, including differential diagnoses, diagnostic tests, final diagnoses, and management strategies according to the patient's age and sex, and case sensitivity.^[1]^

Current research advancements aim to assess further ChatGPT's ability to generate differential diagnoses when prompted with clinical vignettes. In this article, we evaluated ChatGPT's accuracy in generating a preferentially ordered list of differential diagnoses when prompted with patient signs and symptoms of ocular pathologies. Additionally, to assess its accuracy with more particular diagnoses, three categories within ophthalmology were chosen: cornea, retina, and neuro-ophthalmology. The main objective of this study is to determine if ChatGPT-3.5 shows a significant difference in accuracy when generating a list of differential diagnoses in preferential order for these distinct categories with additional risk factors.

##  METHODS

### Study Design

An analysis was conducted to assess the accuracy of potential differential diagnoses generated by ChatGPT-3.5 for a diverse range of ocular pathologies. The study aimed to compare the accuracy of diagnoses generated based solely on signs and symptoms (Method A) with those generated when prompted with additional risk factors, such as age, sex, and past medical history, in addition to the same signs and symptoms (Method B).

### Data Collection

**Figure 1:**
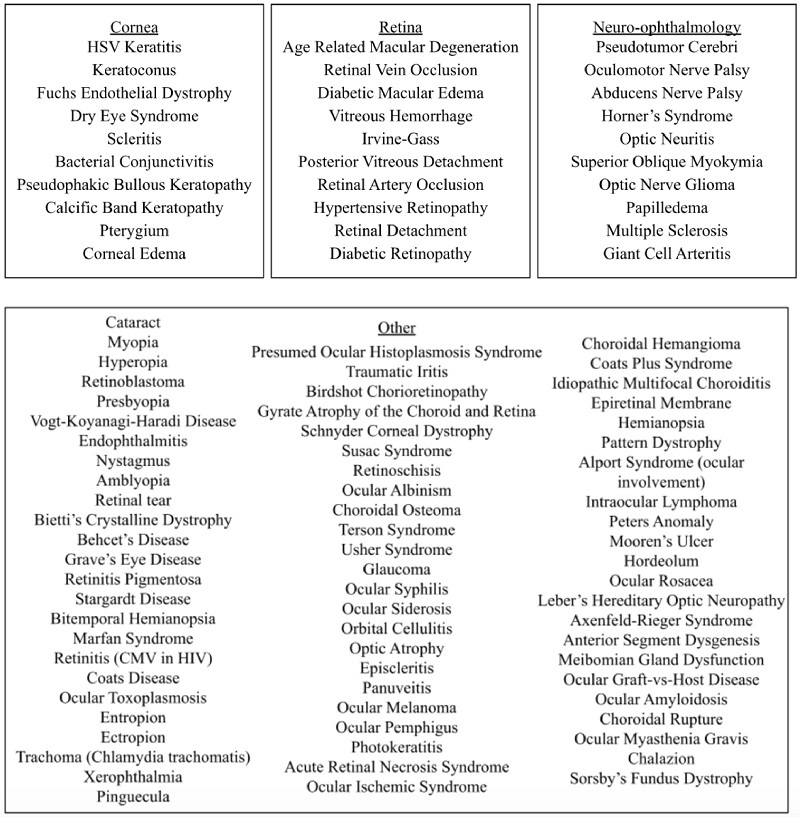
List of 100 ocular diseases inputted into ChatGPT3.5.

A total of 100 ocular pathologies were carefully reviewed and selected [Figure 1]. All signs and symptoms associated with a given pathology were carefully reviewed and extracted from the corresponding EyeWiki article. The signs and symptoms were transferred to an Excel spreadsheet, and then two signs and two symptoms were randomly selected for each clinical scenario. The chosen signs and symptoms were then used to prompt ChatGPT-3.5 to assess whether it could correctly identify the diagnosis.

All sample information was retrieved from the EyeWiki database. The EyeWiki database was trusted as a reliable source, given that any contribution by an ophthalmologist to an EyeWiki article is overseen by an editorial board, editor-in-chief, and deputy editor-in-chief.^[3]^ ChatGPT-3.5 was tasked to output its top four differential diagnoses in preferential order. The full input template for each disease was as follows: “Please list your top four differential diagnosis, in preferential order, based on the following clinical presentation: A patient presents with signs and symptoms of **[input two signs and two symptoms]**.” For example, “A patient presents with signs and symptoms of eyelid retraction, lid lag of upper eye, eye itching, and eye watering.” Using this prompt, ChatGPT-3.5 was able to list thyroid eye disease, allergic conjunctivitis, dry eye syndrome, and conjunctivochalasis. This was denoted as Method A. The output of this process was a list of four differential diagnoses, ranked based on the model's generated probabilities based on prompted input. The accuracy of output was ranked on a scale of 0 to 4. A value of 4 indicated that ChatGPT-3.5 ranked the disease correctly as its first differential, a value of 3 meant it listed the diagnosis as its second highest differential, a value of 2 and 1 suggested a third and fourth differential, respectively, and a value of 0 indicated that ChatGPT-3.5 did not rank the ocular pathology in its list of differential diagnoses at all. Method A was performed on all 100 ocular diseases.

**Figure 2:**
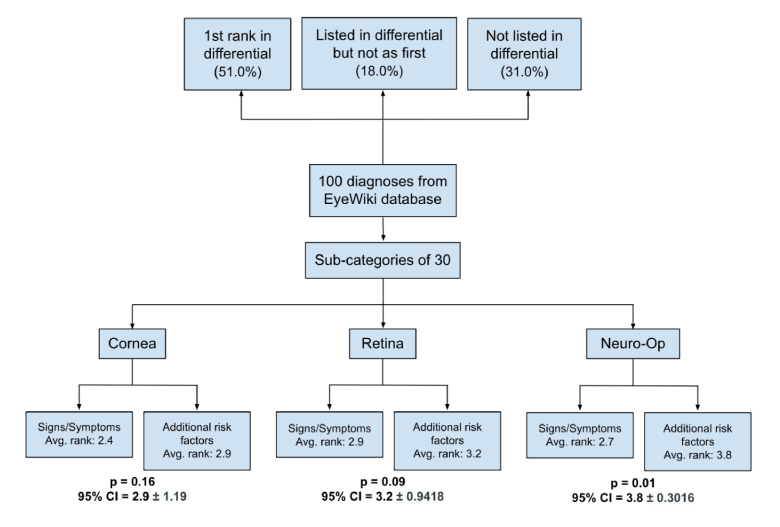
Flowchart of methods.

To further investigate the impact of incorporating additional patient-specific information on ChatGPT-3.5's diagnosis accuracy, 30 of the 100 selected ocular pathologies were subcategorized into three groups based on their primary anatomical involvement: cornea, retina, and neuro-ophthalmology [Figure 2]. The same experiment (Method A) was performed for each ocular pathology in the subcategory. For example, in the cornea subcategory, two signs and two symptoms were inputted into a template and prompted to ChatGPT-3.5 to establish an accurate diagnosis for each disease. The same principle was applied to retina and neuro-ophthalmology subcategories.

**Table 1:** Example display of data collection

**Disease**	**Risk factor: PMH**	**Signs/Symptoms**	**Full clinical vignette input**	**ChatGPT-3.5 output (Results)**
Horner syndrome	Head trauma, carotid dissection, neuroblastoma, thoracic malignancy	Ptosis, ocular redness, nasal stuffiness, headache	A 15-year-old male presents with signs and symptoms of ptosis, ocular redness, nasal stuffiness, and headache. He has a history of head trauma.	Horner syndrome, carotid artery dissection, orbital floor fracture, ocular ischemic syndrome

The second method (Method B) was performed with additional risk factors in addition to the same previous two signs and symptoms prompted in Method A. Method B included the additional risk factors of age, sex, and past medical history extracted from the respective EyeWiki article. This was done across all three subcategories. The full input template for Method B was as follows: “Please list your top four differential diagnoses, in preferential order, based on the following clinical presentation: A **[Age,Sex:M/F]** presents with signs and symptoms of [textbftwo signs and two symptoms]. (He/She) has a history of [textbfPast Medical History]. For example, “A 55-year-old male presents with signs and symptoms of vision loss, eye swelling, a cherry red spot and retinal whitening. The patient has a past medical history of hyperlipidemia.” From this prompt, ChatGPT-3.5 listed central retinal artery occlusion, retinal vein occlusion, central serous chorioretinopathy, and acute angle-closure glaucoma. The same two signs and two symptoms used in Method A were used for Method B. If an EyeWiki article did not specify age as a risk factor, an age from 0 to 100 was selected using a random number generator to avoid selection bias. Similarly, if a disease did not explicitly specify a sex preference, a random number generator was used to select either “male” or “female” randomly. Table 1 demonstrates how ocular pathologies in Method B were prompted into ChatGPT-3.5.

### Statistical Analysis

To assess the accuracy of Methods A and B diagnoses in each subcategory, each dataset was run through a Shapiro-Wilk test to determine whether the data had a normal distribution. The results showed that the data were not normally distributed for each subcategory. As such, a non-parametric one-tailed Wilcoxon signed-rank test was performed to determine the statistical significance between the two groups. The collected data were analyzed using appropriate statistical methods to determine any significant differences in accuracy between Methods A and B. The significance level for all statistical tests was set at *P* < 0.05.

### Ethical Considerations

The data used were obtained from publicly available sources and did not include real patient information. This study was reviewed and approved by the Institutional Review Board (IRB) affiliated with the University of Texas Medical Branch at Galveston Research Regulations and Compliance Office, Galveston, TX, United States. As the study included no actual patient data, the IRB claimed this study as exempt from IRB review and oversight.

##  RESULTS

### Differential Diagnosis Accuracy

**Figure 3:**
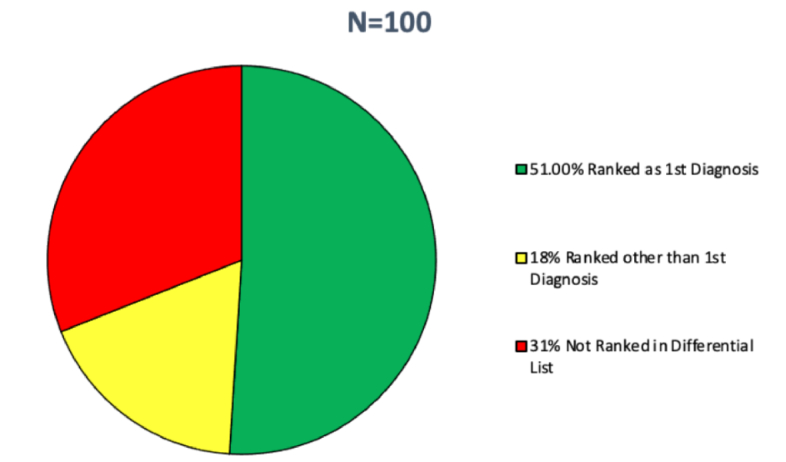
Distribution of ChatGPT's accuracy in diagnosing 100 ocular diseases.

**Figure 4:**
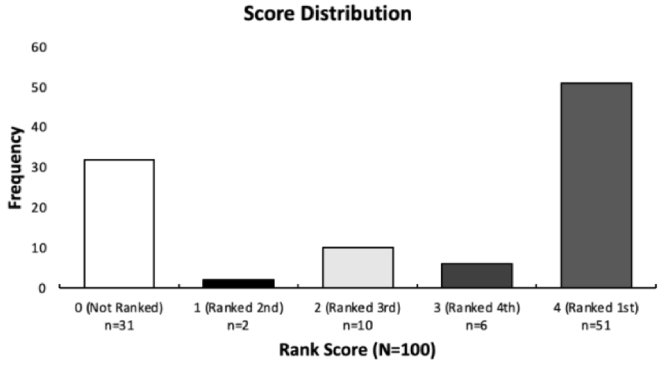
Stratified distribution of rank score across 100 ocular diseases.

The study aimed to assess the accuracy of ChatGPT-3.5 in generating differential diagnoses for a range of ocular pathologies selected from the EyeWiki database. The model's performance was evaluated based on its ability to identify the correct ocular pathology among its suggested list of differential diagnoses. Using Method A for all 100 diseases, ChatGPT-3.5 correctly identified the true diagnosis as its initial differential in 51 cases, resulting in an accuracy rate of 51%. Furthermore, in 18 cases (18%), the model correctly pinpointed the true diagnosis in the differential diagnosis, but not as its initial suggestion; instead, it was ranked as its second, third, or fourth choice. In 31 cases (31%), the model failed to include the true diagnosis within its generated list of potential differentials [Figure 3]. Further analysis of the distribution of rank scores revealed that 2 diseases ranked second, 10 ranked third, and 6 ranked fourth [Figure 4].

### Category-specific Analysis

**Figure 5:**
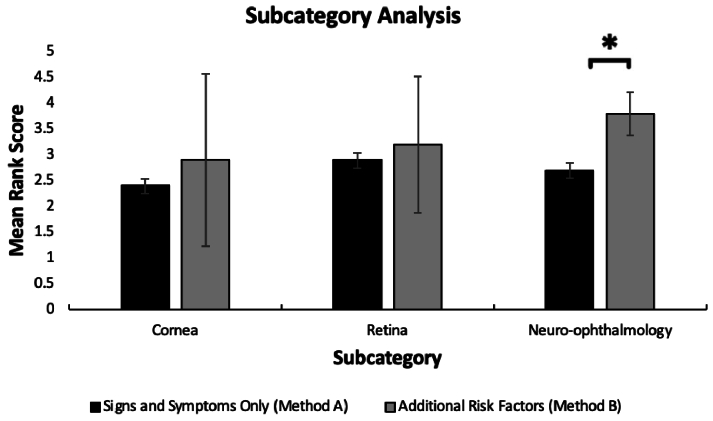
Ocular disease subcategory analysis with and without additional risk factors (Method A vs B).

To determine the impact of incorporating additional risk factors (Method B), each subcategory was compared using Methods A and B. Intriguingly, within the neuro-ophthalmology category, the model exhibited a significantly higher rank value (*P* = 0.01) when prompted with additional rank factors of age, sex, and past medical history (average rank = 3.8) versus when just prompted with two signs and two symptoms (average rank = 2.7) [Figure 5].

In contrast, the cornea (*P* = 0.16) and retina (*P* = 0.09) subcategories did not display statistically significant increases in accuracy when considering additional risk factors compared to diagnoses based solely on two signs and two symptoms.

##  DISCUSSION

### Principal Findings

ChatGPT-3.5 correctly identified a targeted ocular pathology in its list of differential diagnoses in 69% of cases. Furthermore, our results show that ChatGPT-3.5 displayed variations in competency when additional risk factors were incorporated into the clinical vignettes related to the ocular pathologies. Specifically, within the neuro-ophthalmology subcategory, the AI model exhibited a significantly higher rank value (*P* = 0.01) when additional risk factors extracted from the corresponding EyeWiki articles were included in the prompts (average rank = 3.8). This improvement suggests that adding risk factors for ocular pathologies significantly enhanced the ability to generate accurate differential diagnoses. In contrast, the cornea (*P* = 0.16) and retina (*P* = 0.09) subcategories did not show statistically significant increases in accuracy when additional risk factors were included in the clinical vignettes. To the best of our knowledge, this is the first study to evaluate the statistical difference between ChatGPT-3.5's capability to analyze complex versus simple clinical vignettes obtained from an ophthalmology-specific database, EyeWiki.

### Implications and Considerations

The findings of this study highlight the potential utility of integrating patient-specific risk factors into ChatGPT-3.5's input to refine the accuracy of AI-assisted diagnostics for ocular diseases. A previous study assessing ChatGPT's utility within clinical workflow showed that ChatGPT achieved 60.3% accuracy in determining differential diagnoses based on three parameters: history of present illness, results of physical examination, and review of systems. With additional information, such as the results of relevant diagnostic testing, ChatGPT achieved 76.9% accuracy in narrowing a final diagnosis.^[1]^ As previously mentioned, the present study found a significant difference in ChatGPT's diagnostic accuracy using Method B (when prompted with additional risk factors) within the neuro-ophthalmology subcategory, compared to Method A (i.e., inputting only two signs and two symptoms). However, it is important to interpret these results within the limitations of the model's training data and the scope of this study. The traditional clinical decision-making process extends beyond isolated signs, symptoms, and risk factors selected from the EyeWiki database to encompass a holistic understanding of the patient's medical history and context. Nevertheless, this study suggests that AI, specifically ChatGPT-3.5, can be harnessed as a helpful assistant in straightforward medical cases focusing on a single pathology.

AI intends to replicate human thought processes and execution. Similar to a traditional clinical setting where providing more information such as a review of systems, social history, family history, and medical history to a physician will generally allow a more accurate/specific diagnosis, complementary information increases the diagnostic accuracy of ChatGPT. Furthermore, parameters of AI accuracy should assess the substance of the provided information in finalizing a diagnosis. For example, one condition, such as age-related macular degeneration (given that it disproportionately affects a specific age group), may rely more heavily on age rather than sex. Future studies on AI-generated differential diagnoses should consider such factors and could potentially replicate the results, thus assessing AI's ability to differentiate diseases for clinicians.

Several limitations of this study should be acknowledged, including the potential impact of the quality and completeness of randomly selected information from the EyeWiki database on the accuracy of the generated differential diagnoses. Furthermore, the results are based on a single database, which may limit the range of associated risk factors considered for each ocular pathology.

In a recent study, researchers created clinical vignettes for common chief complaints to compare the accuracy of differential diagnosis lists generated by ChatGPT-3 with those provided by physicians. The study showed that ChatGPT-3 achieved a high rate of correct diagnosis (25 out of 30 [83.3%]) of the chief complaint within the five differential-diagnosis lists when prompted by these clinical vignettes. However, the rate of correct diagnosis by physicians remained superior to that of ChatGPT-3 (98.3 vs 83.3%, *P *= 0.03).^[4]^ This observation highlights that AI models like ChatGPT are not yet ready to replace physicians in clinical diagnoses. Instead, they may be utilized more effectively to assist medical providers when interpreting patient data alongside exercising their expertise to reach accurate diagnoses. ChatGPT-3.5 offers valuable insights, even though its design limits extrapolation to complex, real-world clinical scenarios. Additionally, when prompted to evaluate five ophthalmic diseases across eight subspecialties, graded against American Academy of Ophthalmology (AAO) guidelines, ChatGPT assists in patient education but may give incomplete or incorrect information.^[5]^ However, ChatGPT's diagnostic accuracy for glaucoma, when prompted with publicly accessible case studies, matches or surpasses that of senior ophthalmology residents, supporting its potential integration into clinical settings.^[6]^

The accuracy of differential diagnoses generated by ChatGPT relies on the model's training data and may be influenced by its knowledge cutoff date. As for ChatGPT-3.5, its latest knowledge cutoff date is September 2021.^[7]^ This limits the availability of the most recent and accurate information that can influence the knowledge output, discouraging physicians from using ChatGPT as a reliable source to access the most up-to-date information when the AI model generates a differential diagnosis based on the literature. Meanwhile, one recent study found that ChatGPT-4 significantly outperformed ChatGPT-3.5 on 180 Ophthalmic Knowledge Assessment Program (OKAP) practice questions, indicating AI models' rapid development and advancement. This reflects the growing necessity for research into the clinical applications of new AI models.^[8]^ Additionally, the current ChatGPT-3.5 model lacks transparency because a significant portion of its knowledge may be unsupported by evidence-based articles. Studies have shown that ChatGPT model responses lack references, limiting the ability to fact-check the model's outputs and practice evidence-based medicine.^[9]^ One study compared the accuracy of ChatGPT-3.5, ChatGPT-4, and ChatGPT-4 integration with Microsoft Copilot integration on a multiple-choice ophthalmology exam. In this study, ChatGPT-3.5 achieved an accuracy of 59.69%, while Microsoft Copilot achieved a score of 73.60% (*P* < 0.0001). The researchers attributed Copilot's significantly higher correct response rate to its ability to cite reliable sources.^[10]^ Overall, ophthalmologists may not be able to rely entirely on the list of differential diagnoses generated by ChatGPT-3.5 yet. This is due to its unreliability in citing sources, which raises questions about the accuracy of information generated by this AI model.

Future studies that evaluate the role of ChatGPT-3.5 in generating accurate differential diagnoses are recommended to incorporate a larger dataset of ophthalmological diseases and pathologies. This study only assessed 100 ocular pathologies classified into cornea, retina, and neuro-ophthalmology subcategories. An additional research direction could include other subcategories within ophthalmology, such as oculoplastics/orbit and cataract/anterior segment. Doing so would expand the dataset and provide a more comprehensive assessment of ChatGPT-3.5's ability to identify eye diseases correctly.

In summary, given that ChatGPT-3.5 could correctly diagnose ocular diseases based on limited information, this study supports its assistive role as a tool for clinicians in establishing differential diagnoses in ophthalmology. The accuracy of ChatGPT-3.5 in creating a list of differential diagnoses significantly improved by adding risk factors to the clinical vignettes. This was reflected by the results obtained within the neuro-ophthalmology subcategory (*P* = 0.01). However, there was no significant improvement for the two other subcategories (cornea and retina). Future studies may test additional subcategories with varying amounts of clinical information to assess the diagnostic accuracy of this AI model. Further trials should assess the reproducibility and accuracy of AI-generated outputs in more complex cases, such as when prompted with real patient information or when incorporating data from multiple medical databases to consider associated risk factors for medical pathologies. Furthermore, studies comparing diagnoses given by ChatGPT versus physicians are warranted to measure the utility and efficacy of this AI model.

##  Financial Support and Sponsorship

The University of Texas Medical Branch Galveston's Department of Ophthalmology & Visual Sciences financially supported this study.

##  Conflicts of Interest

None.
